# Evaluation of Blue Honeysuckle Berries (*Lonicera caerulea* L.) Dried at Different Temperatures: Basic Quality, Sensory Attributes, Bioactive Compounds, and In Vitro Antioxidant Activity

**DOI:** 10.3390/foods13081240

**Published:** 2024-04-18

**Authors:** Min Yu, Beibei Wang, Zhiqiang Huang, Jinjiao Lv, Yunfei Teng, Tianbo Li, Yu Zhang, Kun Dong, Dong Qin, Junwei Huo, Chenqiao Zhu

**Affiliations:** 1 College of Horticulture & Landscape Architecture/National-Local Joint Engineering Research Center for Development and Utilization of Small Fruits in Cold Regions, Northeast Agricultural University, Harbin 150030, China; yuminzhuzhu@126.com (M.Y.); moncrieffbritany@gmail.com (B.W.);; 2 College of Life Science, Northeast Agricultural University, Harbin 150030, China; 3 Department of Horticulture, Heilongjiang Academy of Agricultural Science, Harbin 150040, China

**Keywords:** haskap, convective drying, total phenolic content, total anthocyanin contents, antioxidant capacity

## Abstract

This study aims to comprehensively investigate the effects of hot-air dehydration on the quality of blue honeysuckle berries (*Lonicera caerulea* L.). The results demonstrated that drying with hot air at 40–65 °C for 7–72 h resulted in blue honeysuckle berries with a moisture content of 0.21–1.10 g H_2_O/g dry weight. Generally, low to medium temperatures (40–55 °C) showed a better effect on the quality than high temperatures (60–65 °C). Specifically, drying at 40 °C exclusively resulted in better retention of cuticular wax, the best sensory appearance, and the highest total phenolic content. Drying at 45 °C and 50 °C resulted in the highest antioxidant capacity and the optimal sensory flavor. Drying at 55 °C led to the highest soluble solid/acid ratio, ascorbic acid concentration, total flavonoid, and total anthocyanin. The work introduces an innovative raw berry product and provides a comprehensive practical and theoretical framework for convective dehydration of blue honeysuckle berries.

## 1. Introduction

Blue honeysuckle (*Lonicera caerulea* L.) is a deciduous perennial shrub native to the cool temperate regions of Eurasia and North America. It produces small berries with an elongated elliptic or cylindrical shape and colors ranging from azure to sapphire depending on the composition of the fruit background color and the intensity of cuticular wax [1]. Blue honeysuckle berry, which has an aromatic, juicy, sour-sweet, and rich flavor profile, is commercially named as ‘Honeyberry’ in Europe and America, ‘Haskap’ in Canada and Japan, and ‘Lan-Dian Guo’ in China [2]. Currently, blue honeysuckle berry has emerged as a novel fruit for its rich flavor and distinctive appearance. Moreover, its exceptional agronomic traits, including remarkable freezing tolerance, ultra-early ripening, and outstanding resistance to pests and diseases, have contributed to the rapid expansion of its cultivation in cold-temperature zones of the Northern Hemisphere, such as northeast China, eastern Europe, Canada, and Hokkaido in Japan. It was estimated that its global cultivation area had reached approximately 10,000 hectares by 2022 [3]. In addition to serving as fresh fruit, blue honeysuckle berry exhibits great potential in food processing as a nutrient supplement owing to its remarkably high concentrations of bio-functional phytocompounds such as phenolics, anthocyanins, flavonoids, and vitamin C [2]. Moreover, due to its abundant and relatively exclusive accumulation of cyanidin-3-glucoside (76–92% of the total anthocyanin content), blue honeysuckle berry has been widely used as a natural source of pigmentation in food processing, particularly in the juice industry [4].

Despite its multiple distinctive advantages, blue honeysuckle berry is confronted with several challenges in both the table and processing markets due to its high vulnerability to rotting during storage and spoilage during transport. These challenges are mainly caused by intrinsically fragile skin and the remarkably tender and succulent texture of the fruit, as well as rapid postharvest senescence [3,5]. Currently, despite the wide adoption of rapid field-heat removal techniques for freshly consumed blue honeysuckle berry, long-distance transport still heavily relies on cold-chain logistics, and the average shelf-life remains shorter than one week, which leads to inevitable losses during transport [6]. For food processing purposes, immediate quick-freezing and subsequent refrigeration storage are indispensable procedures despite the increase in production cost, which is particularly crucial when dealing with berries mechanically harvested or sourced from wild *L. caerulea* var. *edulis* [7]. At present, there is an urgent need for a viable, flexible, easily operated, and cost-effective method to reduce the heavy reliance on cold-chain logistics and refrigerated storage in blue honeysuckle production. Additionally, there is also a need for preprocessed or preliminary-processed products that can serve as an intermediate between fresh and quick-frozen products of blue honeysuckle berry.

Dehydration is an old preservation technique widely adopted for fruits, and dried fruit products derived from specific fruits such as jujube, figs, and goji are even more popular among consumers than their fresh counterparts [8,9]. However, the dehydration of blue honeysuckle berry is faced with significant challenges due to its unique fruit composition characterized by two ovaries enclosed within a copula, resulting in a nested or semi-closed fruit structure (compound accessory fruit). These inherent characteristics make the fruit susceptible to cracking or fracturing during dehydration. Moreover, the fruit is prone to easy abscission and rapid senescence, making it particularly difficult to achieve natural withering and solar dehydration [10]. Also, despite the application of various pretreatments on blue honeysuckle berries, including freezing, thawing, juice extraction, and squeezing, as well as the development of assisted drying methods, such as microwave, osmotic, and freeze drying, these methods heavily rely on equipment, making them impractical for current production practices [11,12,13]. Convective or hot-air drying, which is characterized by simple design, flexible operation, and cost effectiveness, seems to be the optimal solution for the processing and preservation of blue honeysuckle berries [14,15]. However, the drying of blue honeysuckle berries has made limited progress due to the inherent challenges associated with their high moisture content and susceptibility to rotting, as well as the potential negative impacts on dried product quality, such as superficial crust, loss of bioactive compounds, decline in flavor, or undesirable shrinkage [16,17]. Therefore, it is highly necessary to comprehensively investigate the effectiveness of hot-air drying of blue honeysuckle berries and evaluate the quality of the dried products.

To develop a practical hot-air dehydration method for blue honeysuckle berries, create an innovative raw product with diverse potential applications, and offer valuable information for related research and technologies, we investigated the hot-air drying conditions and evaluated the basic qualities, sensory appearance, flavor, total bioactive compounds, and in vitro antioxidant activity of blue honeysuckle berries dried at different temperatures. The results are expected to facilitate the development of dehydration techniques specific for blue honeysuckle berries, as well as provide a viable approach to enhance current production.

## 2. Materials and Methods

### 2.1. Plant Materials

The blue honeysuckle cultivar ‘Lanjingling’ (*Lonicera caerulea* L., CNPVP 20200389) was used as the experimental material (Figure S1) [1]. The plants were cultivated for six years at the Experimental Farm of Northeast Agricultural University (NEAU) in Xiangyang town, Harbin, China (E 126°56′2″, N 45°46′10″). On reaching commercial maturity (SS: TA ≥ 8) [3], the fruits were manually harvested and immediately subjected to a rapid field-heat removal process by the blowing of ambient air at room temperature (~20 °C) in the dark for 30 min. To minimize the removal of cuticular wax, no specific surface cleaning or washing procedures were performed. The samples were subsequently transported to the laboratory in pre-chilled coolers (Excursion 47L, Esky, Sydney, Australia) and stored in an environment of 4 °C and 90% relative air humidity for further treatment and measurement.

### 2.2. Hot-Air Drying Treatment

The drying temperatures were initially set at 35 °C, 40 °C, 45 °C, 50 °C, 55 °C, 60 °C, 65 °C, 70 °C, and 75 °C. For each treatment, approximately 300 berries with normal shape, uniform size, and freedom from pests and diseases were selected. The initial moisture content on the dry basis was determined using a halogen moisture tester (YLS16A-pro, Techcomp, Shanghai, China). The drying treatments were conducted using a fruit dehydration oven (LT-154, Chigo, Foshan, China) equipped with fan-assisted forced-air circulation at a velocity of 2.0 m/s. The oven offered a selectable temperature range from 30 °C to 90 °C, and was operated at a rated power of 1000 kW. The mass changes were measured using an electronic balance (QUINTIX124-1S, Sartorius, Göttingen, Germany) every 1, 2, and 4 h for 65–75 °C, 50–60 °C, and 35–45 °C, respectively, until consecutive measurements showed variations less than 0.10 g. The moisture content (MoC) was expressed as “gram H_2_O of per gram dry mass” (g H_2_O/g DW). The relative moisture ratio (*MoR*) was calculated following the equation reported previously [18,19]: *MoR* = [*M_t_* (dry basis moisture content at a specific time point) − *M_e_* (equilibrium dry basis moisture content)]/[*M*_0_ (initial dry basis moisture content) − *M_e_*].

### 2.3. Basic Quality Determination

The color of the dried berries was measured using a portable color spectrophotometer (CM-700d, KONICA MINOLTA, Tokyo, Japan) with a measuring area of 3.0 mm in diameter with 10° observer and D65 illuminant. The color values were shown in the CIELAB color space system (L*a*b*), in which L* value indicates lightness (1–100), a* indicates green-red deviation (−120–120), and b* indicates blue-yellow deviation (from −120 to 120). For the quantification of soluble solids (SS) and total acid content (TA), 0.50 g of dried berry was finely powdered using a ceramic mortar in liquid nitrogen, and then distilled water was added to a volume of 5.0 mL. The solution was vortexed for 5 min to achieve a homogenized suspension, allowed to stand for an additional 5 min, and, finally, centrifuged at 4000 rpm for 1 min. The total soluble solids (Brix°) and total acid (%) of the supernatant (300.0 μL) were measured using a digital refractometer (PAL-BX|ACID F5 Master Kit, ATAGO, Tokyo, Japan) following the manufacturer’s instructions.

### 2.4. Sensatory Evaluation

A total of twenty panelists, consisting of ten male and ten female volunteers with prior consumption experience of fresh blue honeysuckle berries and aged between 19 and 38 years, were recruited from the students and staff of the College of Horticulture & Landscape Architecture at NEAU. Ethical permission was not required. All participants were informed of all the details of the experiment, and they gave their consent to take part in the sensory study and for the use of their information prior to the start of the sensory experiment. For the sensory description of the appearance and flavor of dried blue honeysuckle berry, two scoring sheets were developed by all co-authors based on relevant studies on raisins [20], dried rabbiteye blueberries [21], dried blackcurrants, and dried sea-buckthorns [22]. The scoring sheets (Tables S1 and S2) comprised three ranks on a 15-point intensity scale, respectively, for six appearance attributes (color, glossiness, wrinkle, integrity, cuticular wax, and comprehensive appearance acceptability) and six flavor attributes (sweetness, sourness, bitterness, astringency, smell, and comprehensive flavor acceptability). The panel underwent a brief 1.5-h training prior to the scoring, during which each test attribute was thoroughly discussed. The reference score of each attribute for fresh berries was deliberated upon, agreed upon, and clearly defined, ensuring comprehensive comprehension of the definition of each attribute by all panelists. Then, mock evaluation of ten dried berries from each drying treatment was conducted in individual degustation booths with white light at the National-Local Joint Engineering Research Center for Development and Utilization of Small Fruits in Cold Regions. The rights and privacy of all panelists were protected following appropriate protocols (National Standard of China: GB/T 43396-2023, GB/T 10220-2012, GB/T 16291.1-2012, and GB/T 23470.2-2009) [23,24,25,26], including voluntary participation, clear disclosure of study requirements, informed consent from participants, no release of participant data without their knowledge, and ability to withdraw from the study at any time.

### 2.5. Phytochemicals Determination

For each phytochemical determination, the sample (dried or fresh samples) was manually pulverized in liquid nitrogen, and subsequently stored at −80 °C until measurement. All determinations were performed in triplicate. The vortex was performed using a micro vortex mixer (MX-S, DLAB, Beijing, China). The ultrasonication was conducted using an ultrasonic bath (SN-QX-100D, SUNNE, Shanghai, China). Centrifugation was carried out using a micro high-speed centrifuge (5425R, Eppendorf, Stevenage, UK). Extract filtrations were performed utilizing 0.45 μm syringe filters (13 mm-PES, BKMAMLAB, Changde, China). The rotary mixing was performed using a tube rotator (MX-RL-E, DLAB, Beijing, China). Spectrophotometric measurements were conducted on a 96-well microplate reader (Synergy™ H1, Biotek, Winooski, VT, USA) following the manufacturer’s instructions.

For total ascorbic acid (AsA) determination, o-phenylenediamine spectrophotometric method was applied [27]. For each extraction, 0.2 g sample powder was mixed with 5.0 mL of an extraction solution consisting of metaphosphoric acid (HPO_3_, 0.4 mol/L) and acetic acid (CH_3_COOH, 2.5%, *v*/*v*); the resulting mixture was vortexed for 1 min and then centrifuged at 10,000 rpm for 1 min. The filtered supernatant was utilized for AsA determination following the protocol described in the National Standard “GB 5009.86—2016” [28]. The fluorescence intensity was measured with excitation and emission wavelengths of 338 and 420 nm, respectively. The result was expressed as “milligram AsA of per gram mass” (mg/g).

The determination of total phenolic content (TpC) was conducted using the Folin–Ciocalteu method [29]. For each extraction, 0.2 g of sample powder was added to a solution containing 5.0 mL methanol/water/HCl (90:10:1, *v*/*v*); the mixture was homogenized by vortexing for 1 min, followed by ultrasonication for 15 min and rotary extraction for 2 h; the mixture was subjected to centrifugation at 5000 rpm for 1 min and the resulting supernatant was filtered for TpC determination. The determination protocol was performed following the Folin–Ciocalteu method modified for small berries [30,31]. The absorbance at 765 nm was measured and gallic acid (C_7_H_6_O_5_) was served as the calibration standard. The results were expressed as “milligram gallic acid equivalent per gram mass” (mg GAE/g).

The total flavonoid content (TfC) was determined using the aluminum chloride method [32]. For each extraction, 0.2 g of sample powder was combined with 5.0 mL of a 60% ethanol solution; the mixture underwent vortexing for 1 min, followed by ultrasonication for 30 min and subsequent centrifugation at 10,000 rpm for 1 min under a temperature of 4 °C. The clarified supernatant was utilized for the determination of TfC in accordance with the established protocols outlined in National Industrial Standard “SN/T 4592—2016” [33], while referencing the modified protocols for chokeberry powder, cranberry juice, and fresh blueberry [34,35]. The absorbance was measured at 415 nm and quercetin (C_15_H_10_O_7_) was utilized as the calibration standard. The results were expressed as “milligram quercetin equivalent per gram mass” (mg QCT/g).

The total anthocyanin content (TaC) was quantified using the pH differential method [36]. For each extraction, a 0.2 g sample was extracted using the TpC extraction procedure. The filtered supernatant was utilized for determination of TaC following a specifically developed protocol for blue honeysuckle [30,37]. The absorbance measurements were taken at 530 nm and 700 nm in potassium chloride buffer (0.025 mol/L, pH 1.0) and sodium acetate buffer (0.4 mol/L, pH 4.5), respectively; cyanidin-3-glucoside (C_21_H_21_ClO_11_) was employed as the calibration standard and the results were expressed as “milligram cyanidin-3-glucoside equivalent per gram mass” (mg C3G/g).

The standard substances (ascorbic acid: A103537, gallic acid: G131992, quercetin: Q111274, and cyanidin-3-glucoside: C498608) were purchased from Shanghai Aladdin biochemical technology Co., Ltd. (Shanghai, China) (https://www.aladdinsci.com/cat-product/chemicals-and-biochemicals.html, accessed on 22 September 2022). The other reagents were sourced from Shanghai Enzyme-linked Biotechnology Co., Ltd. (https://en.mlbio.cn/, accessed on 15 September 2023).

### 2.6. Antioxidant Activity Determination

The antioxidant activity of the dried and fresh blue honeysuckle berries was evaluated using 2,2′-azino-bis-(3-ethylbenzothiazoline-6-sssulfonic acid (ABTS) [38], 2,2-diphenyl-1-picrylhydrazyl (DPPH) [39], and ferric reducing antioxidant power (FRAP) [40] methods. All determinations were performed in triplicate. For the extraction of each measurement, 0.5 g of manually pulverized (in liquid nitrogen) sample powder was mixed into 10.0 mL of methanol/water/HCl solution (90:10:1, *v*/*v*) and then sonicated for 15 min twice and stood for 12 h at 20 °C in darkness. The extract was centrifuged for 5 min (10,000 rpm, 4 °C), and the filtered supernatant was served for antioxidant activity determination following the ABTS, DPPH, and FRAP protocols specifically developed for blue honeysuckle berry [30]. The calibration curves were calculated based on Trolox equivalents and expressed as “μmol Trolox equivalents of per gram mass” (μmol TE/g). All colorimetric assays were conducted on a 96-well microplate reader (Synergy™ H1, Biotek, Winooski, VT, USA) following the manufacturer’s instructions. The chemical reagents were sourced from Sinopharm chemical reagent Co., Ltd. (Shanghai, China) (https://www.reagent.com.cn/, accessed on 20 August 2023).

### 2.7. Statistical Analysis

The raw data obtained from manual records and instruments were entered into Excel 2018 for descriptive statistics analysis. All values were calculated based on three replicates, and the results were presented as mean ± standard deviation. One-way ANOVA was performed using IBM SPSS Statistics for Windows (v. 27.0, IBM Corp., Armonk, NY, USA) with the built-in post hoc test methods of LSD and Duncan. Pearson correlation analysis and principal component analysis (PCA) of the multivariate dataset were performed using Origin 2021 software (v. 9.85, OriginLab Corp., Northampton, MA, USA). Statistical illustrations were generated with the assistance of ChiPlot [41].

## 3. Results and Discussion

### 3.1. Hot-Air Drying Conditions of Blue Honeysuckle Berries

Given the limited information about the convective dehydration of blue honeysuckle berries to date, a wide temperature range (35 °C to 75 °C) was preliminarily selected with an internal increment of 5 °C, and the experimental results are summarized in Table 1. The moisture content (MoC) of fresh blue honeysuckle berries ranged from 6.24 to 7.72 g H_2_O/g dry weight (DW), with a mean value of 7.11 g H_2_O/g DW. The treatments from 40 °C to 75 °C successfully achieved the desired level of dryness within the time ranging from 4 to 72 h, resulting in a final MoC ranging from 0.21 to 1.10 g H_2_O/g DW (mean = 0.52) (Table S3). Due to the relatively high rotting percentage (11.11%) and slow drying progress of 35 °C treatment until 72 h, it was terminated at 72 h. Overall, the MoC of blue honeysuckle berry exhibited an exponential decrease with increasing drying time (Figure S2 and Table S4). Higher drying temperatures significantly reduced the required drying time, and the final MoC of 40–55 °C treatment groups were significantly influenced by the drying temperature (Table S5). Notably, 75 °C and 70 °C treatments resulted in high spoilage ratios (≥50%), which was primarily characterized by bursting within the first two hours of treatment.

The low dehydration efficiency at 35 °C and high spoilage ratio observed under treatments at 70 °C and 75 °C suggest that temperatures lower than 35 °C or higher than 70 °C are not practically or economically viable for the drying of blue honeysuckle berries. Therefore, these three treatments were excluded from further analysis, and the temperature treatments of 40 °C, 45 °C, 50 °C, 55 °C, 60 °C, and 65 °C were designated as LJL-40, LJL-45, LJL-50, LJL-55, LJL-60, and LJL-65 hereafter. To the best of our knowledge, there has been only one study of hot-air drying of blue honeysuckle berries to date, which simply and solely used a wood dryer for the drying process [17]. Compared with their findings, our drying method demonstrated a higher drying efficiency and improved berry dryness, which can be attributed to the use of a specialized fruit dryer equipped with a reticular fruit salver, resulting in the enhancement of the convection speed. However, the dehydration efficiency in the present study was expectedly lower than that obtained by the methods with pretreatment procedures or assistant equipment, and also the methods applied on the powder, juice extraction, or pomace of blue honeysuckle berry [11,12,13,42]. In addition, given the significantly longer processing time required at 40 °C than that required at 65 °C (more than 10-fold), it is necessary to comprehensively assess the economic viability prior to actual production. Moreover, in comparison to the convective dehydration of other small berry crops with similar initial moisture contents, such as blueberry [31], cranberry [43], and wolfberry [44], the hot-air drying process for blue honeysuckle berries requires a longer duration and results in a higher level of dryness. This phenomenon may be attributed to the higher succulence of fresh blue honeysuckle berries, resulting in a relatively lower proportion of bound water content.

### 3.2. Color and Basic Quality

**Figure 1:**
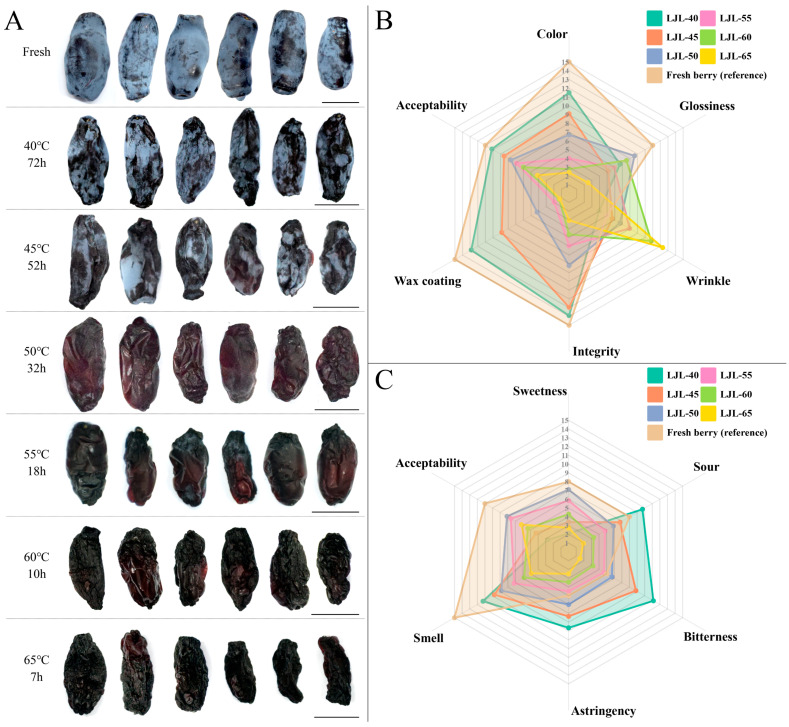
Morphology (**A**), sensory appearance (**B**), and flavor (**C**) evaluation of 40–65 °C hot-air dried blue honeysuckle berries. In (**A**), six randomly selected dried/fresh fruits of each temperature treatment were presented; bar = 1.0 cm. In (**B**,**C**), LJL-40: drying temperature = 40 °C; LJL-45: 45 °C; LJL-50: 50 °C; LJL-55: 55 °C; LJL-60: 60 °C; and LJL-65: 65 °C. The illustration of the temperature treatments was based on the average value scored by a panel of 20 members, the reference standards for each attribute and fresh berry can be found in Tables S3 and S4, and the statistical analysis of the score data is presented in Tables S5 and S6.

From a macroscopic point of view, the hot-air dried blue honeysuckle berries exhibited colors of dark blue, dark purple, or coke black (Figure 1A). Notably, LJL-40 samples displayed conspicuous cuticular wax on the surface, while LJL-45 samples showed a minimal amount of wax, and cuticular wax was nearly negligible in the remaining treatment groups. For all the six treatment groups, the L*, a*, and b* values ranged from 6.99 (LJL-65) to 30.28 (LJL-40), –0.70 (LJL-40) to 3.49 (LJL-50), and –6.32 (LJL-40) to 1.82 (LJL-60), respectively (Table 2). The b* and L* values of all groups exhibited an increasing and decreasing trend in response to rising temperature, respectively, indicating that higher temperatures induce the fading of the blue color and darkening of the overall color.

To assess the basic quality of hot-air dried blue honeysuckle berries, soluble solids (SS) and total acidity (TA) were quantified in a 10-mL water solution containing 1.0 g of dried berry sample. The SS and TA of all the six groups ranged from 4.94 Brix° (LJL-40) to 8.37 Brix° (LJL-65), and from 0.723% (LJL-40) to 1.231% (LJL-65), respectively, exhibiting a gradually increasing trend with rising temperature (Table 2). The SS/TA ratio initially displayed an increasing trend, and reached the peak at LJL-55 (9.67), followed by gradual decreases. Notably, no significant differences were observed in SS, TA, and SS/TA ratio between consecutive temperature treatments from 50 °C to 65 °C, from 40 °C to 55 °C, and from 45 °C to 55 °C, respectively, suggesting that temperatures from 50 °C to 55 °C may represent a temperature range for relatively stable sugar and acid contents.

As reported by Bors [45], it is a great challenge to retain the natural cuticular wax in the dehydration process of blue honeysuckle berries. The hot-air drying method proposed in the present study for the first time achieved a significant retention of cuticular wax (40 °C/72 h) for dried blue honeysuckle berries. Furthermore, it has been well-documented that there is a close correlation between higher temperature exposure and undesirable changes in color, such as darkening, browning, or fading [14]. Hence, it can be inferred that the observed decrease in L* value and increase in b* value were caused by the increase in treatment temperature. However, there was an unexpected peak in a* value at 50 °C, which could be attributed to the complete loss of cuticular wax at this specific temperature or drying time. Moreover, compared with fresh blue honeysuckle berries [1,3], the dried blue honeysuckle berries showed no remarkable enhancement of SS/TA ratio, and relative to other dried small berries [18], the dried blue honeysuckle berries showed a relatively low level value of SS/TA ratio (~10). These results suggested that further dehydration-based processing of blue honeysuckle berry should involve sugaring.

### 3.3. Sensory Evaluation of Appearance and Flavor

To primarily delineate the visual and gustatory attributes of hot-air dried blue honeysuckle berries, a panel comprising 20 individuals with previous consumption experience of fresh blue honeysuckle berry was recruited, and sensory appearance and flavor surveys were conducted based on reference standards using a 15-point scale with three ranks (Tables S3 and S4). The scores of color, wax, and integrity of the samples decreased as the temperature increased, while those of wrinkles exhibited a contrary trend (Figure 1B, Table S6). The score of glossiness showed no discernible pattern in response to temperature changes. The comprehensive ranking of appearance acceptability was LJL-40 (average score of 10.15), LJL-45 (8.55), LJL-50 (7.65), LJL-55 (6.90), LJL-60 (6.00), and LJL-65 (4.15). Notably, only the score of LJL-40 exceeded 10, which can be categorized as “appealing” according to the reference standards, while LJL-60, LJL-55, LJL-50 and LJL-45 received scores within the range of 5 to 10, indicating an “uncertain” appearance. That of LJL-65 was below 5, classifying it as having an “unsavory” appearance.

In terms of flavor (Figure 1C, Table S7), the scores for odor, sourness, astringency, and bitterness demonstrated a decreasing trend with increasing temperature, and no consistent patterns were observed in the score of sweetness with varying temperature. The comprehensive ranking of flavor acceptability was LJL-50 (8.10), LJL-55 (7.60), LJL-65 (6.20), LJL-60 (5.35), LJL-45 (4.30), and LJL-40 (2.75). Although both LJL-50 and LJL-55 received relatively high scores, neither was ranked as “enjoyable” with a score above 10; in addition, despite being scored with “original berry aroma”, both LJL-40 and LJL-45 were classified as having an “unsavory” overall flavor, which might be attributed to their high levels of astringency, bitterness, and sourness.

In the present study, sensory characterization was conducted to preliminarily describe the appearance and flavor. Overall, the panel assigned relatively higher scores to the appearance of the LJL-40 and LJL-45 samples, potentially due to their superior fruit color and cuticular wax retention [46]. Conversely, LJL-50 and LJL-55 were assigned with relatively higher scores for flavor, which might be attributed to their higher sweetness levels and well-balanced taste. Unexpectedly, the acceptability of both appearance and flavor in the six treatment groups was low (4.15–10.15; 2.75–8.10), indicating the nondeterminacy of hot-air dried blue honeysuckle berries as an instant snack and suggesting the application potential of dried blue honeysuckle berry as a raw material in food processing. However, given the intricate nature of sensory attributes and their pivotal roles in determining consumer willingness [47], future research might prioritize a comprehensive and targeted investigation into consumer preferences for dried blue honeysuckle berries, which will facilitate more reasonable determination of their potential to be utilized as a raw material, an ornamental food ingredient, or a flavor additive in the food industry.

### 3.4. Contents of Ascorbic Acid, Phenolics, Flavonoids and Anthocyanins

The ascorbic acid content (AsA), total phenolic content (TpC), total flavonoid content (TfC), and total anthocyanin content (TaC) were measured to further assess the bio-functional compounds of the 40–65 °C hot-air dried blue honeysuckle berries (Table 3). The AsA levels in the six temperature groups ranged from 1.09 mg/g (LJL-65) to 1.50 mg/g (LJL-55), exhibiting an initially increasing and subsequently decreasing trend with rising temperature. The same trends were observed for TfC and TaC. Compared with fresh berries, LJL-40, LJL-45, LJL-50, LJL-55, LJL-60, and LJL-65 showed 50.29%, 54.42%, 57.56%, 58.82%, 64.31%, and 71.07% losses of AsA, respectively (Table S8). The TpC of the six groups ranged from 66.18 mg/g (LJL-65) to 104.96 mg/g (LJL-40), exhibiting a gradually decreasing trend with increasing temperature. Compared with equivalent fresh berries, the six treatment groups displayed 14.71%, 34.03%, 51.03%, 57.97%, 61.74%, and 65.82% losses of TpC, respectively (Table S8). The TfC of the six groups ranged from 18.56 mg/g (LJL-40) to 24.28 mg/g (LJL-55), and declined by approximately 9.74%, 17.42%, 21.05%, 22.50%, 24.10%, and 29.89% during the dehydration process, respectively (Table 3 and Table S8). For TaC, the values ranged from 9.20 mg/g (LJL-65) to 13.99 mg/g (LJL-55), while approximately 31.53%, 41.02%, 45.42%, 48.59%, 58.93%, and 67.27% of TaC were lost during dehydration in the six groups, respectively.

Although AsA, TfC, and TaC reached peaks at LJL-55, no significant differences were observed in the levels of AsA between LJL-50 and LJL-55, the levels of TfC among LJL-45, LJL-50, LJL-55, and LJL-65, or the levels of TaC among LJL-45, LJL-50, and LJL-55 (Table S8). These results suggested that the drying of blue honeysuckle berries at temperatures from 50 °C to 55 °C may result in relatively high levels of all these three compounds in the final products. Significantly, AsA exhibited the highest susceptibility to temperature among the four compounds, and approximately 50–70% of AsA was degraded during dehydration. In contrast, TfC displayed a relatively lower susceptibility to dehydration, and only showed about 10–30% loss during dehydration. These findings are consistent with those reported by Senica, Stampar, Ercisli, Sladonja, Poljuha, and Mikulic-Petkovsek [17]. Furthermore, TpC and TaC are predominant bio-functional compounds in blue honeysuckle berry, and approximately 15–65% and 30–70% of them were degraded after dehydration, which is generally consistent with the results previously reported using different drying methods [7,13]. However, compared with previously reported dried blue honeysuckle berry products, the absolute concentration of the four bio-functional compounds in this study was slightly lower (by approximately 5–20%) [12,42]. This could be attributed to the exclusive use of convective drying without any auxiliary methods and the utilization of fresh and whole fruit as the material without any pretreatment, which might extend the overall drying time and thereby lead to greater degradation of the four compounds [14].

### 3.5. In Vitro Antioxidant Capacity

The in vitro antioxidant capacities of the dried blue honeysuckle berries were evaluated with the DPPH, ABTS, and FRAP methods (Table 4). The DPPH radical scavenging capacity of the six treatment groups ranged from 273.66 μmol TE/g (LJL-65) to 797.67 μmol TE/g (LJL-45), with LJL-45 exhibiting the highest value, followed by LJL-50, LJL-40, LJL-55, LJL-60, and LJL-65. With an increasing drying temperature, 36.40% (LJL-40) to 83.78% (LJL-65) of DPPH capacity was lost compared with that of corresponding fresh berries (Table S9). The ABTS values of the six groups ranged from 589.26 μmol TE/g (LJL-65) to 792.61 μmol TE/g (LJL-45), following the order of LJL-45 > LJL-40 > LJL-50 > LJL-55 > LJL-60 > LJL-65. Compared with corresponding fresh berries, the dried berries showed 68.55% (LJL-40) to 84.25% (LJL-65) losses of ABTS (Table S9). The FRAP of the six groups ranged from 439.23 μmol TE/g (LJL-65) to 584.99 μmol TE/g (LJL-45), followed a descending order of LJL-45 > LJL-50 > LJL-55 > LJL-40 > LJL-60 > LJL-65. Similarly, 72.38% (LJL-40) to 86.10% (LJL-65) of FRAP was lost with increasing temperature (Table S9).

Compared with dried blueberry [21,31], the hot-air dried blue honeysuckle berry in this study showed approximately 30–50% higher in vitro antioxidant capacity, which could be attributed to the inherently greater antioxidant capacity of fresh blue honeysuckle berries [2,30]. Furthermore, the overall antioxidant activity range of DPPH, ABTS, and FRAP (273.66–797.67 μmol TE/g) was closely approximate to that of numerous traditional dried herbal medicines [48], indicating the application potential of dried blue honeysuckle berry for therapeutic purposes beyond natural pigments, flavor additives, and nutrient supplements. Notably, LJL-45 samples showed peaks of all the three indicators, which can be attributed to an equilibrium between the concentration and loss of antioxidants. However, given the overall 35–85% decline in antioxidant capacity, a comprehensive evaluation is imperative to attain an optimal balance between overall antioxidant capacity, antioxidant capacity per unit weight, and the dehydration efficiency for specific application purposes in the future.

### 3.6. Correlation and Principal Component Analysis

**Figure 2:**
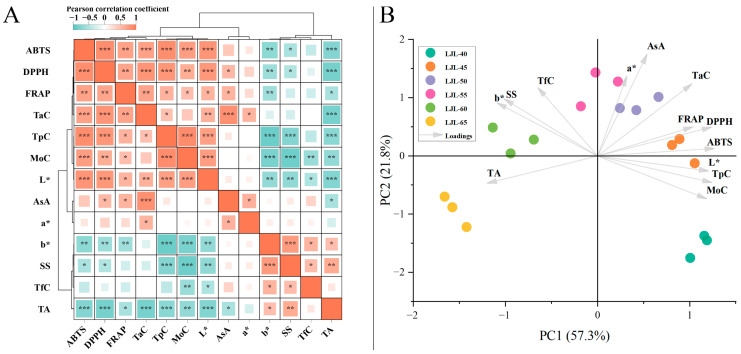
Correlation heatmap (**A**) and principal component bi-plot (**B**) of the quality attributes for hot-air dried blue honeysuckle berries. TfC (total flavonoid content); TaC (total anthocyanin content); AsA (ascorbic acid content); MoC (moisture content); TA (total acid); SS (soluble solids). *: *p* < 0.05; **: *p* < 0.01; ***: *p* < 0.001.

The internal relationship among the quality attributes in hot-air dried blue honeysuckle berries was investigated through correlation analysis (Tables S10 and S11). The quality attributes of the six groups were clearly clustered into two distinct clades (Figure 2A). One clade encompasses ABTS, DPPH, FRAP, TaC, TpC, MoC, L*, AsA, and a*; whereas the other clade comprises b*, SS, TfC, and TA. In terms of the four bio-functional compounds, AsA showed significant positive correlations with DPPH (*r* = 0.54, *p* < 0.05) and FRAP (*r* = 0.57, *p* < 0.05), whereas TpC and TaC demonstrated significant positive correlations with ABTS (*r* = 0.85, *p* < 0.001; *r* = 0.71, *p* < 0.001), DPPH (*r* = 0.79, *p* < 0.001; *r* = 0.81, *p* < 0.001), and FRAP (*r* = 0.58, *p* < 0.05; *r* = 0.69, *p* < 0.01). In addition, MoC demonstrated significant correlations with multiple quality attributes, indicating its potential to serve as both a physical indicator of berry dehydration and an internal quality indicator.

To characterize the six treatment groups of dried blue honeysuckle berries, a principal component analysis (PCA) was conducted based on the measured quality attributes. The two major principal components (PC1 and PC2) were extracted from the multivariate data (Figure 2B and Figure S3). PC1 and PC2 accounted for 57.3% and 21.8% of the total variance, respectively, resulting in a 79.1% cumulative explanation of the total variance. ABTS, TpC, DPPH, TA, b*, and SS exerted significant influence on PC1, while AsA, a*, TaC, MoC, TA, and TpC had substantial impacts on PC2 (Tables S12 and S13). Moreover, the samples exhibited clear separation along the PC1 axis, with LJL-65 and LJL-60 positioned on the left side, while LJL-45 and LJL-40 were situated on the right side; along the PC2 axis, LJL-50 and LJL-55 samples were located above, whereas LJL-40 and LJL-65 were positioned below.

In terms of berry products, previous studies have consistently reported significant positive correlations between bio-functional compounds and antioxidant capacities [14,49]. In this study, TpC and TaC were found to play dominant roles in determining the antioxidant capacity of dried blue honeysuckle berries, as evidenced by their significant associations with DPPH, ABTS, and FRAP. These findings are also consistent with those in previous research on fresh blue honeysuckle berries [2]. However, considering the relatively low concentration and susceptibility to degradation during dehydration of TfC and AsA, as well as variations in their extraction methods employed in this study, further research utilizing a more standardized experimental approach or more precise quantification equipment is necessary to estimate the contribution of AsA and TfC towards the antioxidant activity demonstrated by blue honeysuckle berries. In addition, our PCA analysis revealed distinct quality characteristics between LJL-40 and LJL-65. Specifically, LJL-40 showed exceptional sensory appearance and high retention of both bio-functional compounds and antioxidant capacity, while LJL-65 exhibited the highest dehydration efficiency but the least favorable performance in other aspects. The remaining four groups demonstrated both sequential clustering and differentiation from each other, suggesting that the selection among them should be based on their discrepant quality characteristics and potential specific application objectives.

## 4. Conclusions

In this study, fresh blue honeysuckle berries were successfully dried using hot-air (65–40 °C and 2.0 m/s) for 7–72 h, resulting in dryness levels ranging from 0.21 to 1.10 g H_2_O/g DW. Treatment at 40 °C retained the most cuticular wax. The ranking of treatments in terms of appearance acceptability follows the order of 40 °C, 45 °C, 50 °C, 55 °C, 60 °C, and 65 °C, while that of flavor acceptability follows the order of 50 °C, 55 °C, 65 °C, 60 °C, 45 °C, and 40 °C. The six treatment groups exhibited ascorbic acid contents ranging from 1.09 mg/g (65 °C) to 1.50 mg/g (55 °C), total phenolic content from 66.18 mg/g (65 °C) to 104.96 mg/g (40 °C), total flavonoid content from 18.56 mg/g (40 °C) to 24.28 mg/g (55 °C), and total anthocyanin content from 9.20 mg/g (65 °C) to 13.99 mg/g (55 °C). Their antioxidant capacities were estimated from 273.66 μmol TE/g (65 °C) to 797.67 μmol TE/g (45 °C) using DPPH, from 589.26 μmol TE/g (65 °C) to 792.61 μmol TE/g (45 °C) using ABTS, and from 439.23 μmol TE/g (65 °C) to 584.99 μmol TE/g (45 °C) using FRAP. Correlation analysis revealed significant positive correlations between total phenolic content, total anthocyanin content, and antioxidant capacities. Collectively, blue honeysuckle berries dried at relatively low temperatures (40 °C and 45 °C) exhibited better appearance, retention of bio-functional compounds, and antioxidant capacity. Moderate temperatures (50 °C and 55 °C) tended to improve the flavor and concentration of bio-functional compounds, while high temperatures (60 °C and 65 °C) only resulted in greater dehydration efficiency. Overall, this study provides a practical and viable hot-air drying method for blue honeysuckle berries, essential information for the development of related food processing technologies and products, and valuable clues for research on the dehydration of small berry crops.

## Figures and Tables

**Table 1:** Conditions of hot-air dehydration of blue honeysuckle berry under temperature range of 35–75 °C.

Drying Temperature	Initial MoC (g H_2_O/g DW)	Drying Time	Final MoC (g H_2_O/g DW)	Rotting Percentage	Spoilage Percentage
75 °C/167 °F	7.63 ± 0.90	4 h	0.37 ± 0.08	NA	80.00% ± 13.33%
70 °C/158 °F	7.49 ± 0.75	5 h	0.43 ± 0.16	NA	54.44% ± 15.75%
65 °C/149 °F	6.52 ± 0.61	7 h	0.24 ± 0.03	NA	12.22% ± 5.09%
60 °C/140 °F	6.24 ± 0.77	10 h	0.21 ± 0.07	NA	5.56% ± 5.09%
55 °C/131 °F	7.72 ± 1.43	18 h	0.48 ± 0.19	NA	NA
50 °C/122 °F	7.26 ± 0.88	32 h	0.54 ± 0.20	NA	NA
45 °C/113 °F	7.64 ± 0.92	52 h	0.75 ± 0.14	NA	NA
40 °C/104 °F	7.13 ± 0.93	72 h	1.10 ± 0.19	4.44% ± 1.92%	NA
35 °C/95 °F	6.34 ± 0.80	72 h *	5.10 ± 0.64	11.11% ± 6.93%	NA

MoC: moisture content; *: early termination.

**Table 2:** Color, soluble solid, and acidity of 40–65 °C hot-air dried blue honeysuckle berries.

Sample	L*	a*	b*	SS (Brix°)	TA (%)	SS:TA
LJL-40	30.28 ± 4.33 a	−0.70 ± 1.01 d	−6.32 ± 2.64 d	4.94 ± 0.31 d	0.723 ± 0.081 c	6.90 ± 0.82 b
LJL-45	22.54 ± 4.41 b	1.09 ± 1.01 b	−4.22 ± 1.80 c	6.88 ± 0.58 c	0.785 ± 0.066 c	8.81 ± 1.26 a
LJL-50	25.43 ± 5.10 b	3.49 ± 1.42 bc	−1.58 ± 1.05 b	7.44 ± 1.16 b	0.794 ± 0.068 c	9.40 ± 1.37 a
LJL-55	16.06 ± 2.28 c	1.70 ± 1.59 a	0.80 ± 1.15 a	7.96 ± 0.15 ab	0.826 ± 0.050 c	9.67 ± 0.59 a
LJL-60	10.64 ± 3.58 d	0.21 ± 1.46 cd	1.82 ± 0.96 a	8.18 ± 0.56 a	0.932 ± 0.044 a	8.79 ± 0.68 b
LJL-65	6.99 ± 2.59 e	−0.41 ± 1.73 d	0.63 ± 0.84 a	8.37 ± 0.34 a	1.231 ± 0.065 b	6.82 ± 0.44 b
Fresh pulp	38.81 ± 7.61	0.17 ± 0.35	−9.65 ± 5.19	15.19 ± 0.19	1.83 ± 0.87	8.48 ± 1.42

Values are show in mean ± standard deviation. SS: soluble solids; TA: total acid. The SS and TA of dried samples were measured from 1.0 g DW/10.0 mL water solution. Different letters of dried samples in a same column indicate significant difference (*p* < 0.05).

**Table 3:** Bio-functional compound concentrations of 40–65 °C hot-air dried blue honeysuckle berries.

Sample	AsA (mg/g)	TpC (GAE mg/g)	TfC (QCT mg/g)	TaC (C3G mg/g)
LJL-40	1.19 ± 0.92 d	104.96 ± 7.86 a	18.56 ± 2.58 b	12.23 ± 0.16 b
LJL-45	1.39 ± 0.31 bc	103.50 ± 6.87 a	21.65 ± 3.74 ab	13.43 ± 0.36 a
LJL-50	1.41 ± 0.15 ab	84.17 ± 2.76 b	22.67 ± 2.26 ab	13.62 ± 0.41 a
LJL-55	1.50 ± 0.51 a	78.81 ± 3.96 bc	24.28 ± 1.38 a	13.99 ± 0.36 a
LJL-60	1.32 ± 0.19 c	73.05 ± 4.76 cd	24.21 ± 2.93 a	11.38 ± 0.88 c
LJL-65	1.09 ± 0.26 e	66.18 ± 3.57 d	22.68 ± 1.34 ab	9.20 ± 0.24 d
Fresh pulp	0.62 ± 0.30	31.98 ± 4.44	5.34 ± 0.73	4.64 ± 0.05

Values are mean ± standard deviation of three replications. Value unit of dried samples was expressed as “mg/g dry weight”, while for fresh samples it was expressed as “mg/g fresh weight”. Values of dried berry samples followed with different letters in a same column indicate significantly different at *p <* 0.05 level.

**Table 4:** Antioxidant capacities of 40–65 °C hot-air dried blue honeysuckle berries.

Sample	DPPH (μmol TE/g)	ABTS (μmol TE/g)	FRAP (μmol TE/g)
LJL-40	682.16 ± 68.48 b	748.14 ± 21.14 ab	554.58 ± 74.14 ab
LJL-45	797.67 ± 28.31 a	792.61 ± 28.05 a	584.99 ± 38.16 a
LJL-50	690.29 ± 39.55 b	717.49 ± 30.17 b	580.14 ± 32.42 a
LJL-55	614.91 ± 67.49 b	701.03 ± 29.87 b	561.16 ± 79.03 ab
LJL-60	456.51 ± 74.54 c	611.53 ± 37.22 c	462.51 ± 55.56 bc
LJL-65	273.66 ± 64.70 d	589.26 ± 34.30 c	439.23 ± 70.35 c
Fresh pulp	278.72 ± 14.18	618.15 ± 60.02	521.76 ± 28.39

Values are mean ± standard deviation of three replications. Value unit of dried samples was expressed as “μmol TE/g dry weight”, while for fresh samples it was expressed as “μmol TE/g fresh weight”. TE: Trolox equivalent. Values of dried berry samples followed with different letters in a same column indicate significantly different at *p* < 0.05 level.

## Data Availability

The original contributions presented in the study are included in the article/Supplementary Material, further inquiries can be directed to the corresponding author.

## Supplementary Materials

The following supporting information can be downloaded at: https://www.mdpi.com/article/10.3390/foods13081240/s1: Figure S1. Blue honeysuckle (*Lonicera caerulea* L.) cultivar ‘Lanjingling’; Figure S2. Drying curves of blue honeysuckle berries under different temperatures; Figure S3. Scree plot in PCA analysis; Table S1. Reference standards for sensory appearance evaluation; Table S2. Reference standards for sensory flavor evaluation; Table S3. Additional summary of hot-air dehydration of blue honeysuckle berry under temperature range of 35–75 °C; Table S4. Records of relative moisture content during the dehydration process of blue honeysuckle berries; Table S5. ANOVA statistics of for MoC and relative MoC of treatment groups of 40 °C to 75 °C; Table S6. Statistics of sensory appearance evaluation; Table S7. Statistics of sensory flavor evaluation; Table S8. Loss of bifunctional compounds of hot-air dried blue honeysuckle berries; Table S9. Loss of antioxidant activities of hot-air dried blue honeysuckle berries; Table S10. *R* value matrix of the quality attributes of the dried blue honeysuckle berries; Table S11. *P* value matrix of the quality attributes of the dried blue honeysuckle berries; Table S12. Loading statistics of the attributes in PCA analysis; and Table S13. Score report of the samples in PCA analysis.
