# Supplementary material for: Evaluation of Blue Honeysuckle Berries (Lonicera caerulea L.) Dried at Different Temperatures: Basic Quality, Sensory Attributes, Bioactive Compounds, and In Vitro Antioxidant Activity
Source: Foods. 2024 Apr 18;13(8):1240. doi: 10.3390/foods13081240 (PMC11048952; doi:10.3390/foods13081240)
Supplement: Supplementary file 1 [file foods-13-01240-s001.zip › foods-2946104-supplementary.pdf]

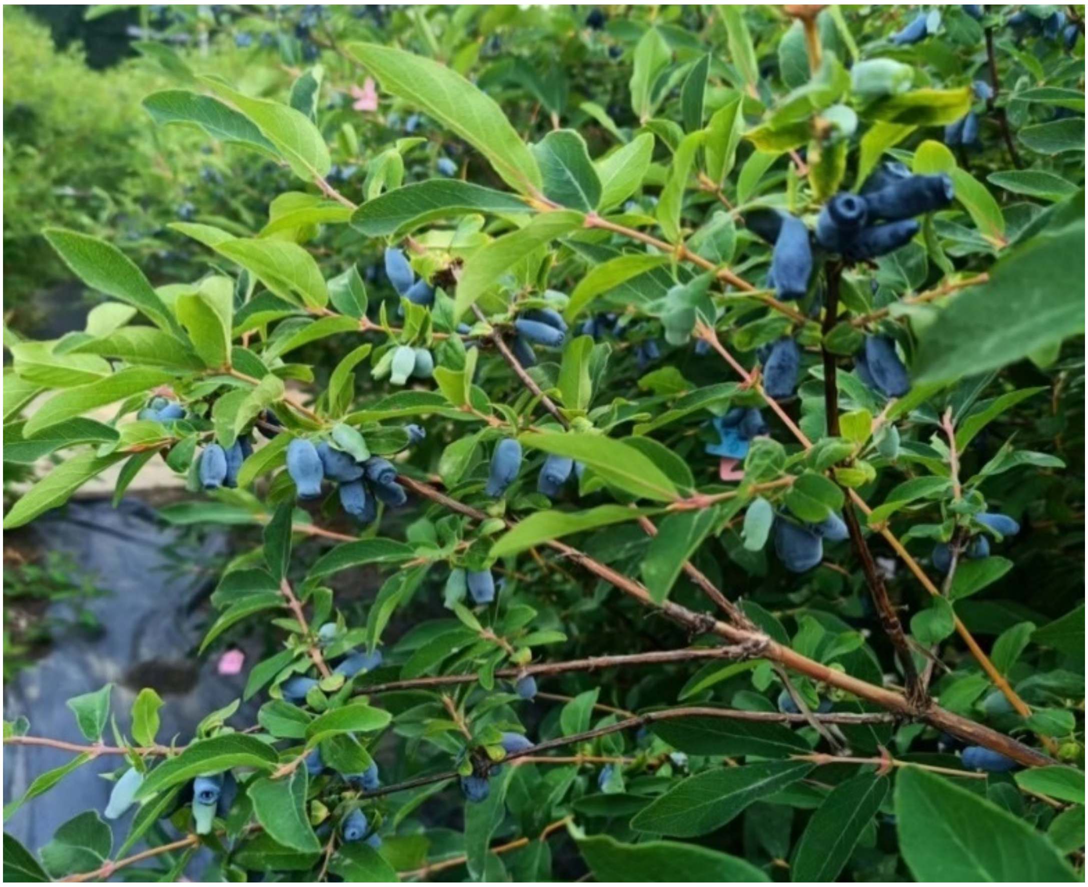

Figure S1. Blue honeysuckle (*Lonicera caerulea* L.) cultivar ‘Lanjingling’ (by Min Yu; July, 2023)

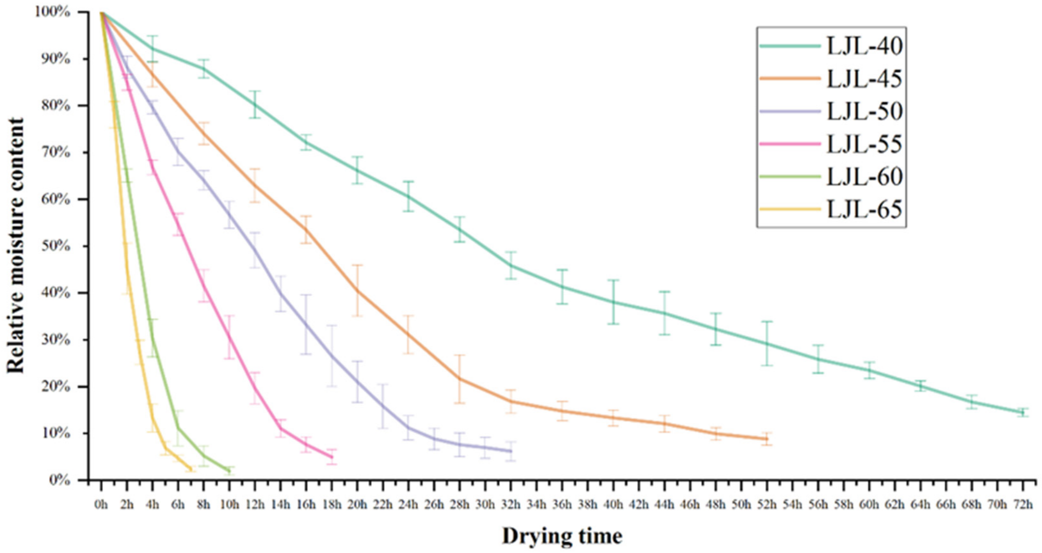

Figure S2. Drying curves of blue honeysuckle berries under different temperatures

LJL-40: 40℃, LJL-45: 45℃, LJL-50: 50℃, LJL-55: 55℃, LJL-60: 60℃, LJL-65: 65℃.

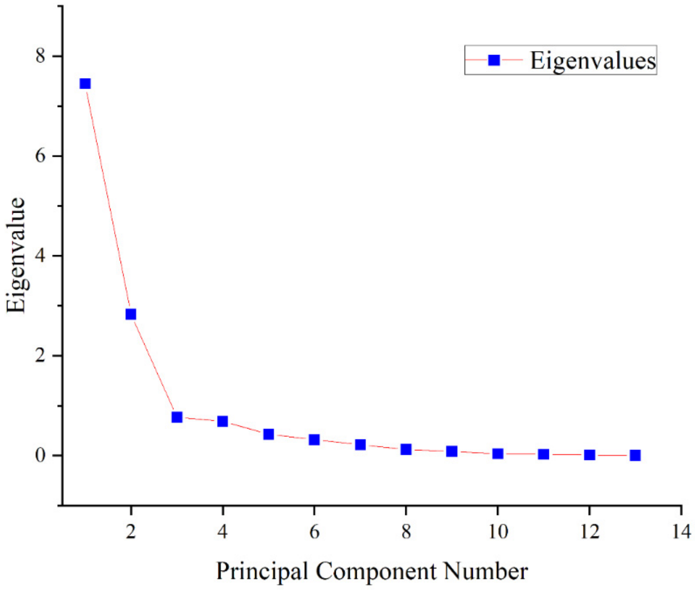

Figure S3. Scree plot in PCA analysis

Table S1. Reference standards for sensory appearance evaluation

Reference	Berry original color	Glossiness	Wrinkle	Integrity	Wax coating	Acceptability
11–15	–Blue	–Glossy	–Heavily	–Compact and intact	–Thick	–Appealing
6–10	Purple black–dark-blue	–Moderate–	–Moderate–	–Moderate–	–Moderate–	–Uncertain–
1–5	Black–	Lackluster–	Slightly–	Fragmentary–	Negligible–	Inedible–
Fresh berry	15	11	5	15	15	11

Table S2. Reference standards for sensory flavor evaluation

Reference	Sweetness	Sour	Bitterness	Astringency	Smell	Acceptability
11–15	–High	–Tart	–High	–No	–Original berry aroma	–Enjoyable
6–10	–Medium–	–Medium–	–Medium–	–Medium–	–Uncertain smell–	–Acceptable–
1–5	Low–	No–	No–	High–	Unpleasant odor–	Unsavory–
Fresh berry	8	8	5	5	15	11

Table S3. Additional summary of hot-air dehydration of blue honeysuckle berry under temperature range of 35–75℃

Drying temperature	Initial MoC (%)	Drying time	Final MoC (%)
75℃/167°F	88.34% ± 1.17%	4 h	4.14% ± 0.31%
70℃/158°F	88.16% ± 1.02%	5 h	4.53% ± 0.28%
65℃/149°F	86.65% ± 1.05%	7 h	4.33% ± 0.46%
60℃/140°F	86.08% ± 1.54%	10 h	4.55% ± 0.61%
55℃/131°F	86.98% ± 3.09%	18 h	4.46% ± 0.72%
50℃/122°F	87.80% ± 1.32%	32 h	5.06% ± 0.14%
45℃/113°F	88.34% ± 1.28%	52 h	5.79% ± 0.71%
40℃/104°F	87.59% ± 1.39%	72 h	7.56% ± 0.46%
35℃/95°F	86.28% ± 1.42%	*72 h	20.39% ± 0.67%

MoC: moisture content.

Table S4. Records of relative moisture content during the dehydration process of blue honeysuckle berries

Time	40℃	45℃	50℃	55℃	60℃	65℃
0h	1.00 ± 0.00	1.00 ± 0.00	1.00 ± 0.00	1.00 ± 0.00	1.00 ± 0.00	1.00 ± 0.00
1h	NA	NA	NA	NA	NA	0.78 ± 0.03
2h	NA	NA	0.88 ± 0.02	0.85 ± 0.02	0.65 ± 0.01	0.45 ± 0.05
3h	NA	NA	NA	NA	NA	0.27 ± 0.03
4h	0.92 ± 0.03	0.87 ± 0.03	0.80 ± 0.01	0.67 ± 0.02	0.30 ± 0.04	0.13 ± 0.03
5h	NA	NA	NA	NA	NA	0.07 ± 0.01
6h	NA	NA	0.70 ± 0.03	0.55 ± 0.02	0.11 ± 0.04	0.05 ± 0.01
7h	NA	NA	NA	NA	NA	0.02 ± 0.01
8h	0.88 ± 0.02	0.74 ± 0.02	0.64 ± 0.02	0.42 ± 0.03	0.05 ± 0.02	NA
10h	NA	NA	0.57 ± 0.03	0.31 ± 0.05	0.02 ± 0.01	NA
12h	0.8 ± 0.03	0.63 ± 0.04	0.49 ± 0.04	0.20 ± 0.03	NA	NA
14h	NA	NA	0.40 ± 0.04	0.11 ± 0.02	NA	NA
16h	0.72 ± 0.02	0.53 ± 0.03	0.33 ± 0.06	0.08 ± 0.02	NA	NA
18h	NA	NA	0.27 ± 0.06	0.05 ± 0.02	NA	NA
20h	0.66 ± 0.03	0.40 ± 0.05	0.21 ± 0.04	NA	NA	NA
22h	NA	NA	0.16 ± 0.05	NA	NA	NA
24h	0.61 ± 0.03	0.31 ± 0.04	0.11 ± 0.03	NA	NA	NA
26h	NA	NA	0.09 ± 0.02	NA	NA	NA
28h	0.54 ± 0.03	0.22 ± 0.05	0.08 ± 0.03	NA	NA	NA
30h	NA	NA	0.07 ± 0.02	NA	NA	NA
32h	0.46 ± 0.03	0.17 ± 0.02	0.06 ± 0.02	NA	NA	NA
36h	0.41 ± 0.04	0.15 ± 0.02	NA	NA	NA	NA
40h	0.38 ± 0.05	0.13 ± 0.02	NA	NA	NA	NA
44h	0.36 ± 0.05	0.12 ± 0.02	NA	NA	NA	NA
48h	0.32 ± 0.03	0.10 ± 0.01	NA	NA	NA	NA
52h	0.29 ± 0.05	0.09 ± 0.01	NA	NA	NA	NA
56h	0.26 ± 0.03	NA	NA	NA	NA	NA
60h	0.23 ± 0.02	NA	NA	NA	NA	NA
64h	0.20 ± 0.01	NA	NA	NA	NA	NA
68h	0.17 ± 0.01	NA	NA	NA	NA	NA
72h	0.14 ± 0.01	NA	NA	NA	NA	NA

Table S5. ANOVA statistics of for MoC and relative MoC of treatment groups of 40°C to 75°C

Drying temperature	Final MoC (g H2O/g DW)	Final relative MoC (%)
75°C/167°F	0.37 ± 0.08 de	0.91 ± 0.70 d
70°C/158°F	0.43 ± 0.16 de	4.61 ± 1.57 c
65°C/149°F	0.24 ± 0.03 e	2.39 ± 0.54 d
60°C/140°F	0.21 ± 0.07 e	1.93 ± 0.85 d
55°C/131°F	0.48 ± 0.19 de	4.96 ± 1.53 c
50°C/122°F	0.54 ± 0.20 cd	6.21 ± 1.98 c
45°C/113°F	0.75 ± 0.14 b	8.82 ± 1.40 b
40°C/104°F	1.10 ± 0.19 a	14.46 ± 0.83 a

Different letters indicate significant difference ($p < 0.05$).

Table S6. Statistics of sensory appearance evaluation

Drying temperature	Color	Glossiness	Wrinkle	Integrity	Wax	Acceptability
40℃	11.50 ± 1.88 a	6.70 ± 1.45 bc	3.95 ± 1.82 e	13.90 ± 0.97 a	12.85 ± 1.35 a	10.15 ± 3.59 a
45℃	9.10 ± 2.57 b	5.15 ± 1.35 d	5.70 ± 2.47 d	12.95 ± 1.47 b	8.85 ± 1.73 b	8.55 ± 3.53 bc
50℃	6.70 ± 1.75 c	8.60 ± 2.28 a	6.70 ± 2.11 cd	8.20 ± 1.88 c	4.20 ± 1.67 c	7.65 ± 3.12 cd
55℃	4.00 ± 1.56 d	5.95 ± 1.90 cd	7.95 ± 2.50 c	5.95 ± 1.39 d	1.95 ± 0.89 d	6.90 ± 2.10 cd
60℃	2.80 ± 1.40 e	7.55 ± 1.67 b	10.80 ± 1.82 b	4.65 ± 1.35 e	1.30 ± 0.57 d	6.00 ± 2.49 de
65℃	2.40 ± 1.10 e	2.50 ± 0.83 e	12.30 ± 1.72 a	3.05 ± 1.05 f	1.20 ± 0.41 d	4.15 ± 2.91 e

Table S7. Statistics of sensory flavor evaluation

Drying temperature	Sweetness	Sourness	Bitterness	Astringency	Smell	Acceptability
40℃	2.10 ± 1.17 e	9.70 ± 2.79 a	11.15 ± 3.34 a	8.65 ± 3.38 a	11.25 ± 2.00 a	2.75 ± 1.62 e
45℃	3.40 ± 1.39 cd	6.75 ± 2.31 b	8.85 ± 2.48 b	7.35 ± 1.73 ab	9.75 ± 2.31 ab	4.30 ± 2.52 de
50℃	7.10 ± 2.27 a	5.90 ± 2.17 bc	5.70 ± 2.36 c	6.00 ± 1.86 b	8.85 ± 2.68 b	8.10 ± 3.40 a
55℃	5.85 ± 1.79 b	4.80 ± 1.47 c	4.65 ± 2.23 c	4.50 ± 2.33 c	7.15 ± 2.90 c	7.60 ± 3.38 ab
60℃	4.30 ± 1.69 c	3.30 ± 1.30 d	3.20 ± 1.82 d	3.45 ± 2.14 cd	5.85 ± 2.54 cd	5.35 ± 2.70 cd
65℃	2.75 ± 1.07 de	1.95 ± 0.76 e	1.50 ± 0.69 e	2.50 ± 1.54 d	4.90 ± 2.69 d	6.20 ± 2.44 bc

Table S8. Loss of bifunctional compounds of hot-air dried blue honeysuckle berries

Treatment	Fresh weight (g)	Dry weight (g)	Index	Loss of AsA	Loss of TpC	Loss of TfC	Loss of TaC
LJL-40	30.93	8.04	3.85	50.29%	14.71%	9.74%	31.53%
LJL-45	31.24	6.37	4.91	54.42%	34.03%	17.42%	41.02%
LJL-50	30.74	5.72	5.37	57.56%	51.03%	21.05%	45.42%
LJL-55	30.21	5.15	5.86	58.82%	57.97%	22.50%	48.59%
LJL-60	32.11	5.38	5.97	64.31%	61.74%	24.10%	58.93%
LJL-65	31.17	5.15	6.05	71.07%	65.82%	29.89%	67.27%

Note: The levels of AsA, TpC, Tfc, and TaC in fresh blue honeysuckle berries were 0.62 mg/g, 31.98 mg/g, 5.34 mg/g, and 4.64 mg/g respectively; the loss of these compounds was calculated using the formula: {1 – [value of dried berry / (Index × value of fresh berry)]} × 100%. The values of the dried berries are shown in Table 3.

Table S9. Loss of antioxidant activities of hot-air dried blue honeysuckle berries

Treatment	Fresh weight (g)	Dry weight (g)	Index	Loss of DPPH	Loss of ABTS	Loss of FRAP
LJL-40	30.93	8.04	3.85	36.40%	68.55%	72.38%
LJL-45	31.24	6.37	4.91	41.66%	73.86%	77.15%
LJL-50	30.74	5.72	5.37	53.92%	78.40%	79.31%
LJL-55	30.21	5.15	5.86	62.37%	80.66%	81.66%
LJL-60	32.11	5.38	5.97	72.56%	83.43%	85.15%
LJL-65	31.17	5.15	6.05	83.78%	84.25%	86.10%

Note: the DPPH, ABTS, and FRAP of fresh blue honeysuckle berry were 278.72 umol TE/g, 618.15 umol TE/g, and 521.76 umol TE/g respectively; the loss of these activities was calculated using the formula: {1 – [value of dried berry / (Index × value of fresh berry)]} × 100%. The values of the dried berries are shown in Table 4.

Table S10. *R* value matrix of the quality attributes of the dried blue honeysuckle berries

rValue	MoC	SS	TA	L*	a*	b*	AsA	TpC	TfC	TaC	DHHP	ABTS	FRAP
MoC	1	-0.8739	-0.6745	0.7388	0.0737	-0.8176	0.0035	0.8623	-0.6390	0.4149	0.6222	0.7417	0.4910
SS	-0.8739	1	0.6368	-0.6927	0.1510	0.7495	0.1588	-0.7623	0.5077	-0.2949	-0.5062	-0.5393	-0.3139
TA	-0.6745	0.6368	1	-0.7710	-0.2858	0.4939	-0.5588	-0.7205	0.1911	-0.8281	-0.8341	-0.7624	-0.5477
L*	0.7388	-0.6927	-0.7710	1	0.1144	-0.6707	0.1555	0.7197	-0.5229	0.5917	0.7863	0.8073	0.5678
a*	0.0737	0.1510	-0.2858	0.1144	1	0.1331	0.5882	0.0052	0.1054	0.5692	0.3300	0.1873	0.2755
b*	-0.8176	0.7495	0.4939	-0.6707	0.1331	1	0.0676	-0.8840	0.5756	-0.2357	-0.6089	-0.7033	-0.5993
AsA	0.0035	0.1588	-0.5588	0.1555	0.5882	0.0676	1	0.1792	0.3378	0.8342	0.5360	0.4201	0.5726
TpC	0.8623	-0.7623	-0.7205	0.7197	0.0052	-0.8840	0.1792	1	-0.4322	0.4793	0.7915	0.8482	0.5793
TfC	-0.6390	0.5077	0.1911	-0.5229	0.1054	0.5756	0.3378	-0.4322	1	-0.0088	-0.2101	-0.3877	-0.3220
TaC	0.4149	-0.2949	-0.8281	0.5917	0.5692	-0.2357	0.8342	0.4793	-0.0088	1	0.8076	0.7122	0.6910
DHHP	0.6222	-0.5062	-0.8341	0.7863	0.3300	-0.6089	0.5360	0.7915	-0.2101	0.8076	1	0.8497	0.6831
ABTS	0.7417	-0.5393	-0.7624	0.8073	0.1873	-0.7033	0.4201	0.8482	-0.3877	0.7122	0.8497	1	0.7050
FRAP	0.4910	-0.3139	-0.5477	0.5678	0.2755	-0.5993	0.5726	0.5793	-0.3220	0.6910	0.6831	0.7050	1

Table S11. *P* value matrix of the quality attributes of the dried blue honeysuckle berries

**pValue**	MoC	SS	TA	L*	a*	b*	AsA	TpC	TfC	TaC	DHHP	ABTS	FRAP
MoC	1	0.000002	0.002139	0.000461	0.771400	0.000034	0.989104	0.000004	0.004306	0.086904	0.005832	0.000427	0.038521
SS	0.000002	1	0.004487	0.001440	0.549647	0.000343	0.529227	0.000235	0.031485	0.234909	0.032059	0.020893	0.204540
TA	0.002139	0.004487	1	0.000180	0.250215	0.037231	0.015922	0.000744	0.447540	0.000022	0.000017	0.000235	0.018634
L*	0.000461	0.001440	0.000180	1	0.651252	0.002317	0.537899	0.000758	0.025989	0.009685	0.000109	0.000051	0.013976
a*	0.771400	0.549647	0.250215	0.651252	1	0.598498	0.010247	0.983683	0.677188	0.013691	0.181041	0.456649	0.268491
b*	0.000034	0.000343	0.037231	0.002317	0.598498	1	0.789811	0.000001	0.012432	0.346349	0.007320	0.001130	0.008571
AsA	0.989104	0.529227	0.015922	0.537899	0.010247	0.789811	1	0.476896	0.170380	0.000017	0.021857	0.082602	0.013002
TpC	0.000004	0.000235	0.000744	0.000758	0.983683	0.000001	0.476896	1	0.073259	0.044153	0.000091	0.000009	0.011759
TfC	0.004306	0.031485	0.447540	0.025989	0.677188	0.012432	0.170380	0.073259	1	0.972230	0.402764	0.111903	0.192481
TaC	0.086904	0.234909	0.000022	0.009685	0.013691	0.346349	0.000017	0.044153	0.972230	1	0.000051	0.000912	0.001495
DHHP	0.005832	0.032059	0.000017	0.000109	0.181041	0.007320	0.021857	0.000091	0.402764	0.000051	1	0.000008	0.001781
ABTS	0.000427	0.020893	0.000235	0.000051	0.456649	0.001130	0.082602	0.000009	0.111903	0.000912	0.000008	1	0.001085
FRAP	0.038521	0.204540	0.018634	0.013976	0.268491	0.008571	0.013002	0.011759	0.192481	0.001495	0.001781	0.001085	1

Table S12. Loading statistics of the attributes in PCA analysis

Attributes	Loadings				
	PC1	PC2	PC3	PC4	PC5
MoC	0.31494	-0.22053	0.07240	-0.23243	0.25978
SS	-0.26958	0.2929	0.24115	0.33073	-0.12204
TA	-0.31927	-0.14011	0.29013	0.27388	0.19144
L*	0.32094	-0.07746	0.01721	-0.16108	-0.50960
a*	0.08409	0.40872	0.51622	-0.48643	0.44209
b*	-0.29011	0.27314	-0.02851	-0.29953	-0.37541
AsA	0.14281	0.52247	-0.05751	0.18019	0.02303
TpC	0.33072	-0.13673	-0.18447	0.12052	0.35055
TfC	-0.17232	0.35054	-0.65485	0.05911	0.33830
TaC	0.27245	0.37108	-0.02549	-0.09962	-0.19084
DPPH	0.32956	0.14875	-0.12920	0.03998	-0.04328
ABTS	0.33761	0.03884	-0.01178	0.17570	-0.09349
FRAP	0.27510	0.14969	0.31811	0.56252	0.00280

Table S13. Score report of the samples in PCA analysis

Samples	Scores				
	PC1	PC2	PC3	PC4	PC5
LJL-40-1	1.15779	-1.37108	-0.76635	1.56796	0.65973
LJL-40-2	1.18671	-1.44691	-0.82016	-1.45149	0.26685
LJL-40-3	1.00489	-1.75064	0.71954	-1.28756	-1.38328
LJL-45-1	0.80780	0.18860	-0.65089	-0.39503	-0.14719
LJL-45-2	0.88841	0.29215	-1.45705	1.14072	0.16369
LJL-45-3	1.05343	-0.12387	1.58512	1.24529	1.26777
LJL-50-1	0.41976	0.78865	1.45166	0.69166	-1.86075
LJL-50-2	0.24088	0.82233	-0.17372	-0.88407	0.48908
LJL-50-3	0.66127	1.01560	0.82878	-1.22961	1.19969
LJL-55-1	-0.18209	0.85413	-1.04874	-0.29107	-1.51390
LJL-55-2	0.22037	1.27894	1.18969	0.32759	0.78094
LJL-55-3	-0.02326	1.43529	-0.61983	0.41801	-0.13958
LJL-60-1	-0.69322	0.28135	0.65909	-0.32890	-1.46346
LJL-60-2	-0.94444	0.04381	-1.22902	0.40164	-0.57777
LJL-60-3	-1.13817	0.48966	-1.15463	-0.56811	1.10586
LJL-65-1	-1.42257	-1.21984	0.66269	1.45435	-0.27345
LJL-65-2	-1.57661	-0.87984	-0.02553	0.48694	0.52229
LJL-65-3	-1.66095	-0.69834	0.84935	-1.29832	0.90348
